# Supplementary material for: Evaluation on Structural Properties and Performances of Graphene Oxide Incorporated into Chitosan/Poly-Lactic Acid Composites: CS/PLA versus CS/PLA-GO
Source: Polymers (Basel). 2021 Jun 2;13(11):1839. doi: 10.3390/polym13111839 (PMC8199591; doi:10.3390/polym13111839)
Supplement: Supplementary file 1 [file polymers-13-01839-s001.zip › polymers-1137640-supplementary.pdf]

Supplementary data

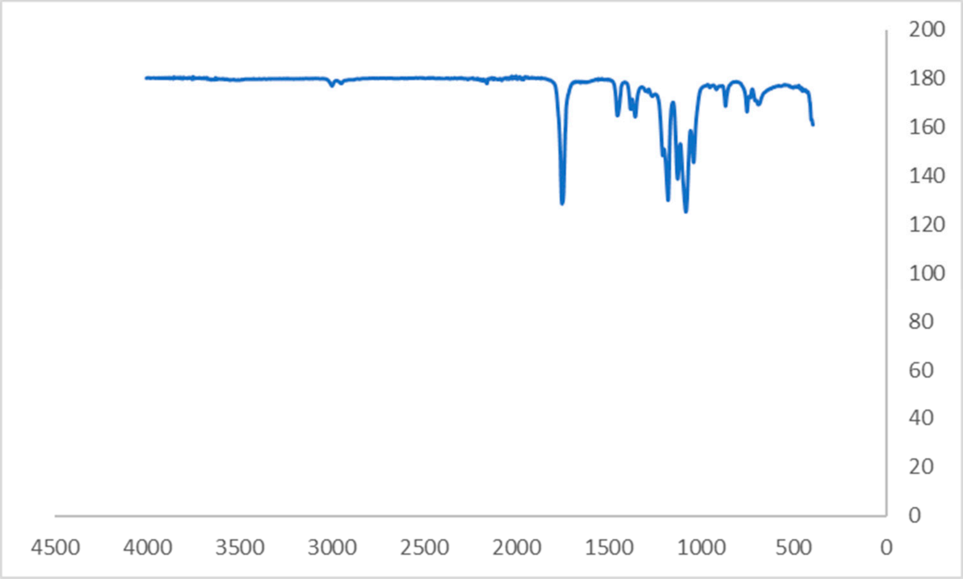

Figure S1. FTIR of PLA sample.

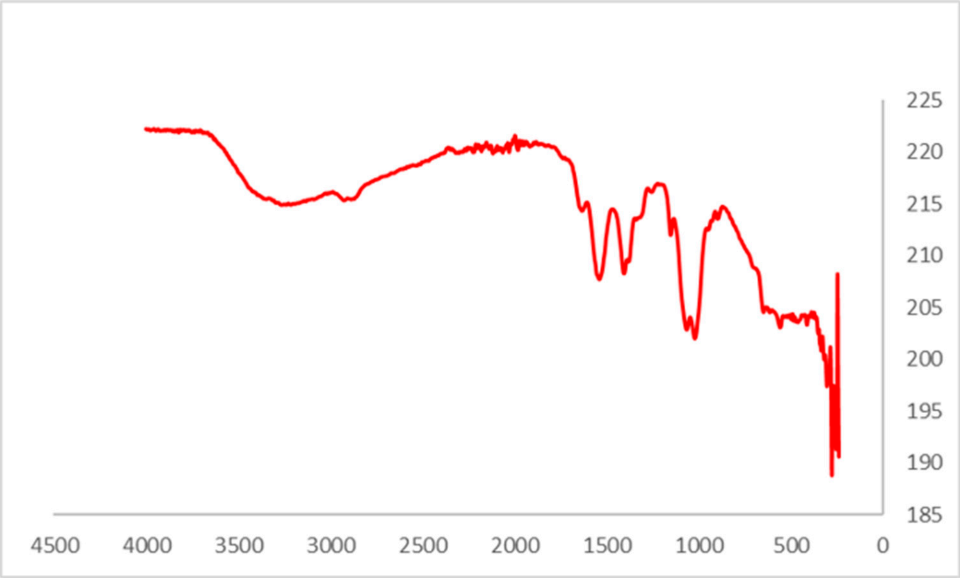

Figure S2. FTIR of DSC sample.

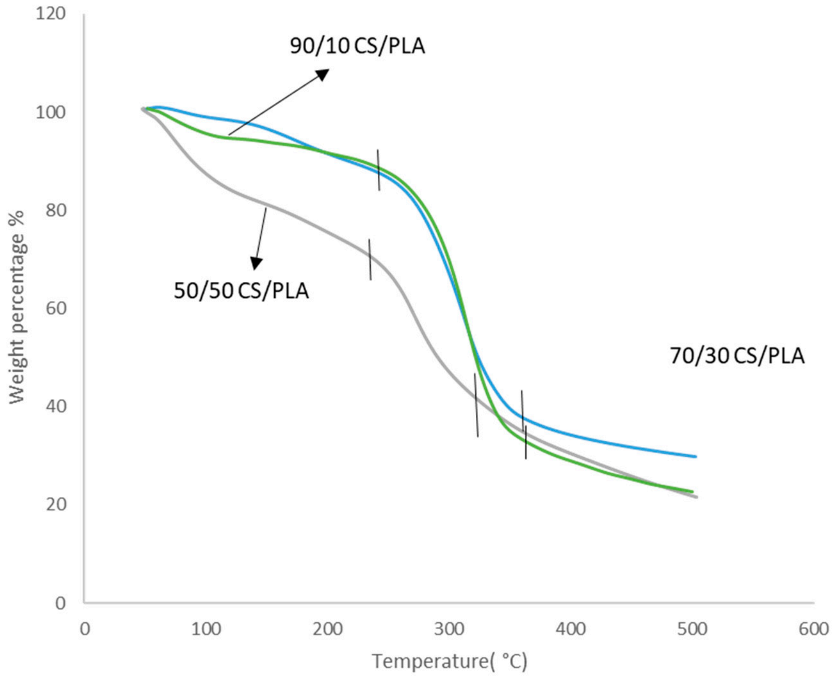

Figure S3. FTIR of CS/PLA without GO samples at different concentration.

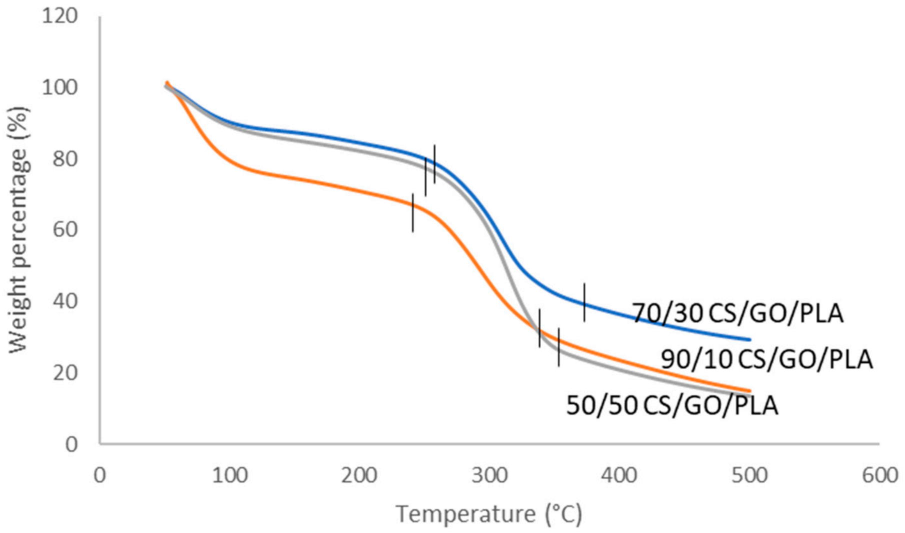

Figure S4. FTIR of CS/PLA with GO samples at different concentration.
